# New Prostate Cancer Targets for Diagnosis, Imaging, and Therapy: Focus on Prostate-Specific Membrane Antigen

**DOI:** 10.3389/fonc.2018.00653

**Published:** 2018-12-21

**Authors:** Alessia Cimadamore, Monica Cheng, Matteo Santoni, Antonio Lopez-Beltran, Nicola Battelli, Francesco Massari, Andrea B. Galosi, Marina Scarpelli, Rodolfo Montironi

**Affiliations:** ^1^Section of Pathological Anatomy, School of Medicine, United Hospitals, Polytechnic University of the Marche Region, Ancona, Italy; ^2^Department of Radiology and Imaging Sciences, Indiana University School of Medicine, Indianapolis, IN, United States; ^3^Oncology Unit, Macerata Hospital, Macerata, Italy; ^4^Department of Pathology and Surgery, Faculty of Medicine, Cordoba, Spain; ^5^Division of Oncology, S. Orsola-Malpighi Hospital, Bologna, Italy; ^6^Institute of Urology, School of Medicine, United Hospitals, Marche Polytechnic University, Ancona, Italy

**Keywords:** prostate cancer, prostate-specific membrane antigen, PSMA, small molecule inhibitors, RNA aptamer conjugates, PSMA-based immunotherapy, PSMA-targeted prodrug therapy, positron emission tomography

## Abstract

The rising incidence rate of the cancer in the prostate gland has increased the demand for improved diagnostic, imaging, and therapeutic approaches. Prostate-specific membrane antigen (PSMA), with folate hydrolase and carboxypeptidase and, internalization activities, is highly expressed in the epithelial cells of the prostate gland and is strongly upregulated in prostatic adenocarcinoma, with elevated expression correlating with, metastasis, progression, and androgen independence. Recently, PSMA has been an active target of investigation by several approaches, including the successful utilization of small molecule inhibitors, RNA aptamer conjugates, PSMA-based immunotherapy, and PSMA-targeted prodrug therapy. Future investigations of PSMA in prostate cancer (PCa) should focus in particular on its intracellular activities and functions. The objective of this contribution is to review the current role of PSMA as a marker for PCa diagnosis, imaging, and therapy.

## Introduction

Prostate-specific membrane antigen (PSMA) is a type 2 integral membrane glycoprotein with folate hydrolase and carboxypeptidase, and internalization activities. This internalization capability is increased up to 3-fold when PSMA is linked to anti-PSMA antibodies. PSMA expression is highest in prostate tissue (secretory acinar epithelium), but detectable levels of PSMA protein are also found in the kidney (proximal tubules), the small bowel (i.e., jejunal brush border), neuroglia (Schwann cells and astrocytes), and salivary glands (1, 2). Notably, PSMA is highly expressed in prostate cancer cells and the vessels of various non-prostatic solid tumors (it is not expressed in the normal vasculature) (3).

With the rise and evolution of several targeted approaches to examine prostate cancer using PSMA, the aim of this contribution is to review the current role of PSMA as a marker for PCa diagnosis, imaging, and therapy.

## Expression And Role Of Psma In Pca

**Figure 1:**
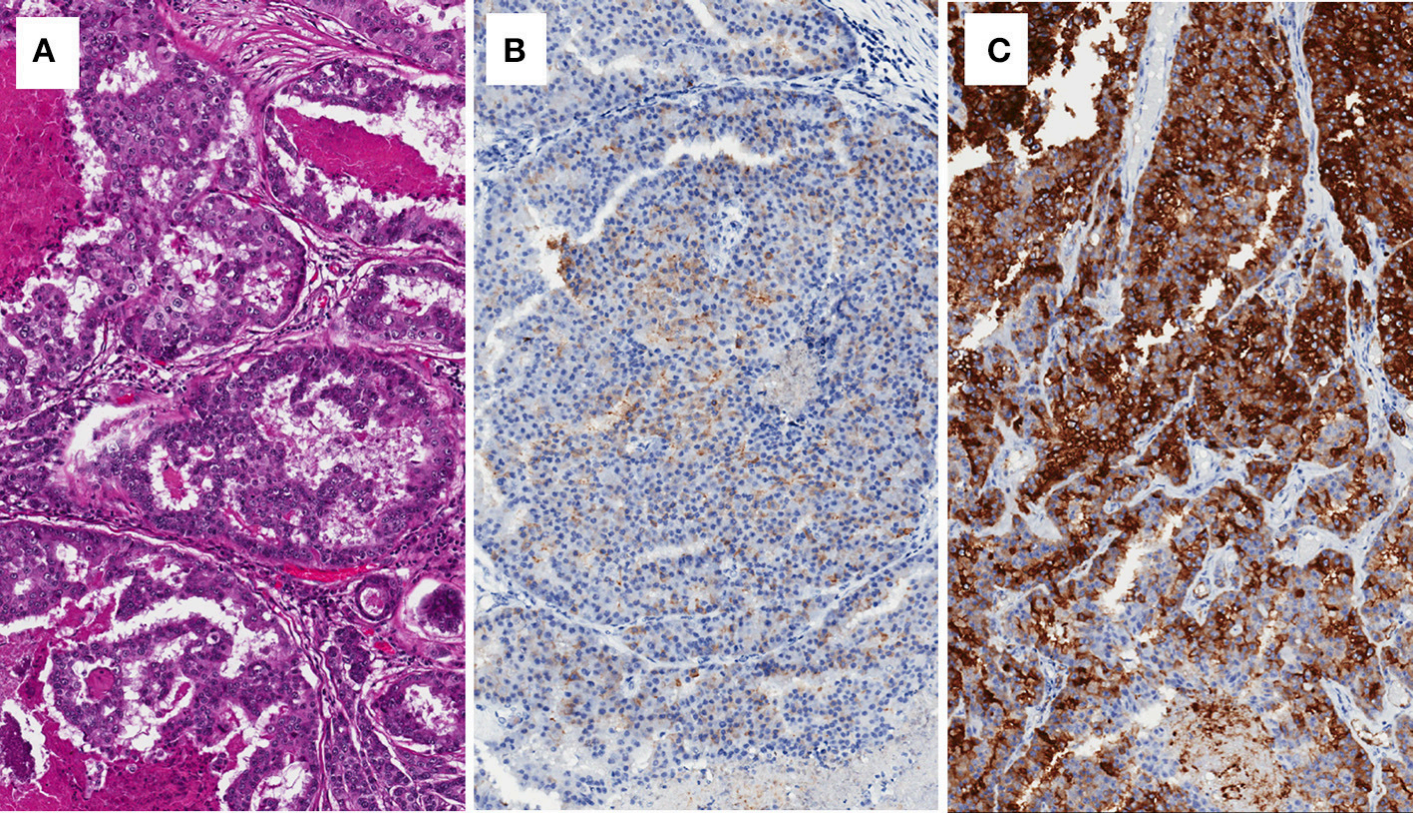
Brain metastasis of prostate cancer with cribriform pattern **(A)**, showing low expression of PSA **(B)**, and intense expression of PSMA **(C)**.

PSMA was originally discovered using the monoclonal antibody 7E11 obtained from the cell membrane of the LNCaP cell line (4). It has been shown by immunohistochemistry that expression of PSMA at the tissue level increases through the progression from normal prostate cells to high-grade prostatic intraepithelial neoplasia (HGPIN) and to PCa (3) (Figure 1). There exists a strong positive correlation between PSMA expression and Gleason score. Elevated PSMA expression is strongly correlated with a high serum PSA. These indications are associated with increased tumor angiogenesis and lack of ets-related gene (ERG) expression which leads to reduced vitamin D and androgen receptor expression (5). PSMA expression is regulated by the androgen receptor (AR). PSMA expression increases dramatically during androgen-deprivation therapy (6).

Downregulation of PSMA expression by AR may be associated to the presence of an enhancer region although no androgen response elements have been identified (7).

PSMA expression is significantly correlated with prostate growth and differentiation (8). In particular, *in vitro* expression of PSMA is associated with an increased cellular folate content. This induces a proliferative property to cells expressing PSMA (9, 10). In addition, PSMA stimulates PCa cell proliferation, migration and survival through the phospho-p38 (P-p38) MAPK pathway in LNCaP cancer cells (11). Guo et al. demonstrated that PSMA knockdown in a LNCaP cell line was associated with not only the inhibition of the pathway of phosphatidylinositol 3-kinase/Akt signaling but also decreased cell proliferation, migration and survival (12).

PSMA is involved in the development of PCa metastases. Xu et al. evaluated four prostate cancer cell lines (i.e., DU145, LNCap, PC-3, and 22RV1) for metastasis-related genes potentially involved in PCa metastasis regulated by PSMA. In their study, *CDH6, MMP3*, and *MTSS1* were seen as PSMA-related genes. Their expression was inversely related with the stage of cancer, thus suggesting their possible involvement in the suppression of PCa metastasis by PSMA (13).

## PSMA-Based Imaging In Patients With PCa

Conventional imaging techniques, such as ultrasound, CT, bone scintigraphy and Magnetic Resonance Imaging (MRI), are at present utilized to detect primary PCa and its metastatic deposits. However, the limitation of such traditional imaging techniques and modalities is their low sensitivity in the detection of recurrent or/and metastatic PCa. Improved imaging modalities are needed to optimize the management of the patients with PCa.

Positron Emission Tomography (PET) and single photon emission computed tomography (SPECT) with emerging radiopharmaceuticals provide more accurate staging for primary cancer, detection of metastatic disease, and restaging of tumor recurrence. PSMA has received considerable attention as a useful marker for imaging purposes in patients with PCa (14, 15). Several PSMA-based approaches have been developed, including antibodies, nanobodies, and small molecule inhibitors.

### Antibodies and Nanobodies

Indium-111 capromab pendetide (^111^In-capromab, ProstaScint®) was the first monoclonal antibody against PSMA used in PCa immunoscintigraphy. Correlation of scan results with tissue specimens showed that ^111^In-capromab detected soft tissue metastases, with an average negative predictive value of 70%, sensitivity of 60%, and positive predictive value of 60% (16–18). However, ^111^In-capromab lacks sensitivity because it recognizes an intracellular epitope of PSMA, thereby targeting only apoptotic/necrotic or damaged cells.

Unlike ^111^In-capromab, J591 is an antibody against the extracellular domain of PSMA. ^111^In-labeled J591 has been evaluated against conventional imaging techniques in the evaluation of bone metastases. ^111^In-labeled J591 identifies 93.7% of skeletal lesions detected by a conventional imaging technique. Thirteen out of Eighteen bone deposits detected only with ^111^In-labeled J591 were successively confirmed to be metastases (19). In a more recent study, J591 has been radiolabeled with 89Zr (20) and 64Cu (21) for PET imaging and demonstrate robust targeting of skeletal, nodal and soft tissue metastasis (22).

A new strategy in the development of high-contrast nuclear imaging is the utilization of specific antibody fragments, called nanobodies. Nanobodies contain antibody-derived smaller fragments (typically the variable domain alone of heavy chain antibodies) that largely retain the specific antigen binding properties of the original antibodies, but with more rapid pharmacokinetics and lower immunogenic potential. Evazalipour et al. compared the properties of different nanobodies radiolabeled with 99 m-Technetium (99 mTc) in PSMA^+^ LNCaP and PSMA^−^ PC3 cell lines and in PSMA^−^ and PSMA^+^ tumor-bearing xenografts through SPECT/micro-CT imaging and tissue analysis. Among the evaluated molecules, nanobody PSMA30 resulted in an important compound for future applications in PCa imaging trials (23).

Interesting results were also obtained with minibodies, i.e., IAB2M, an 80-kDa minibody genetically engineered from the parent antibody J591 that targets the extracellular domain of PSMA. A phase I dose-escalation study in patients with metastatic prostate cancer demonstrated PET imaging with 89Zr-Df-IAB2M is feasible and well tolerated, and targets both bone and soft-tissue disease (24).

### Small Molecules

The identification of the functional (25) and structural (26) homology between N-acetylaspartylglutamate peptidase or NAAALDASE (for which a number of enzymatic inhibitors had been identified) (27, 28) and PSMA has been a major step forward for the development of PSMA-targeted radiotracers. Generally, small molecule PSMA inhibitors consist of zinc binding compounds linked to a glutamate isostere or glutamate. Phosphonate-, phosphate-, and phosphoramidates (1) and ureas (2) constitute the two main families of compounds. Based on NAALADASE homology, several compounds have been developed and labeled with 123I (20, 29, 30), 99mTc (21, 31), 18F (32), 111In (33), and 68Ga (34).

123I-MIP-1072 and 123I-MIP-1095 were the first small molecule inhibitors of PSMA adopted in the clinic. SPECT/CT using these compounds showed a rapid detection of PCa deposits in the bone, soft tissue, and prostate gland of men with metastatic PCa (35). A phase I trial on 131I-MIP-1095 in men with mCRPC is now active (NCT03030885).

Among the emerging PSMA small molecule inhibitors, *N*-(*N*-((*S*)-1,3-dicarboxypropyl) carbamoyl)-4-(18F)fluorobenzyl-L-cysteine (18F-DCFBC) is under evaluation in several ongoing studies. Using 18F-DCFBC, PSMA^+^ PC-3 PIP xenografts were early visualized with little radioactivity in the PSMA^−^ isogenic PC-3 flu xenografts. After 2 h, the PC-3 PIP xenografts remained visible, with clearance of background radioactivity from kidneys, liver and blood (36, 37).

The use of 18F-DCFBC has been investigated in a few patients with Gleason scores between 7 and 9 and with radiological evidence of metastatic PCa. Bone scans or CT identified 21 lesions (5 bone and 16 lymph node lesions), while 32 lesions were visible with 18F-DCFBC PET. Ten of Eleven additional lesions were located in the bone and were suggestive of early bone deposits, indicating the potential of 18F-DCFBC PET in this subpopulation (38). Currently, the use of 18F-DCFBC PET/CT is under evaluation in a study enrolling patients scheduled for surgical prostate (Group 1), or with biochemical recurrence after surgery or radiotherapy (Group 2), or in metastatic PCa patients (Group 3) (NCT02190279). In addition, another ongoing phase I/II study is assessing the potential of 18F-DCFBC PET in the detection of primary PCa, nodal and bone metastases in men at initial diagnosis (NCT01496157) (Table 1).

**Table 1:** Current completed trial on PSMA-based imaging.

**NCT Identifier**	**Study phase**	**Tracer and technique**	**Study outcomes**
NCT01496157	Phase 1 Phase 2	18F-DCFBC PET	PET detection of primary and sextant localization of PCa and detection of metastatic disease at initial staging
NCT02048150	Phase 1	MDX1201-A488	Assess the best dose given before robotic assisted laparoscopic RP to aid in visualization of the prostate
NCT02151760	Phase 1	18F-DCFPyL	Compare diagnostic accuracy of 18-DCFPyL to CT and bone scintigraphy for the detection of metastatic PCa
NCT01667536	Phase 2	99mTc-MIP-1404	Assessment of the diagnostic accuracy in detection PCa within the prostate and metastatic PCa
NCT03558711	Phase 1	18F-PSMA	Safety of administration
NCT02611882	Phase 1 Phase 2	Ga-68 HBED-CC PSMA	SE and SP for the detection of nodal metastasis in high-risk pre-RP patients, of metastatic disease in patients with BCR after RP or radiation therapy, comparison to conventional imaging in CRPC patients.
NCT03486886	Not Applicable	PSMA -PET/CT	Evaluation of detection Yield performance and reproducibility in mPCa Patients
NCT02796807	Phase 2	68Ga-HBED-CC-PSMA PET/CT	Correlation Between SUV on 68Ga-HBED-CC-PSMA and GS in PCa
NCT02488070	Phase 1 Phase 2	68Ga-PSMA PET/CT	Average SUVmax of Ga68 PSMA Uptake Outside the Expected Normal Biodistribution
NCT01359189	Phase 1	ProxiScan	Evaluation of a Transrectal Scintigraphic Detector (ProxiScanTM) for Detection of Primary PCa
NCT02918357	Phase 2 Phase 3	Ga-68 labeled PSMA-11 PET	Sensitivity and PPV of for the detection of metastases a per-patient and per-region-basis confirmed by histopathology
NCT02916537	Phase 1	CTT1057 PET/MR	Evaluation of the safety, pharmacokinetics, and [18F] radiation dosimetry
NCT00712829	Phase 1	123-I-MIP-1072 123-I-MIP-1095	Evaluating the safety, pharmacokinetics, tissue distribution
NCT01615406	Phase 1	99mTc MIP 1404	Comparison study of 99mTc-MIP-1404 (SPECT)/CT imaging to histology
NCT01261754	Phase 1	99mTc-MIP-1404 and MIP-1405	Safety, pharmacokinetics, biodistribution in mPCa patients; newly diagnosed, high-risk PCa and healthy subjects
NCT01572701	Phase 1	99mTc-MIP-1404	Measure activity counts in tissue samples post-surgery, Intensity of 99mTc-MIP-1404 Uptake with Respect to PSMA expression in Men With PCa Undergoing RP and/or PLND
NCT02190279	Early phase 1	18F-DCFBC PET/CT	Assess the ability to identify sites of localized, recurrent and metastatic PCa
NCT00992745	Phase 1	123-I-MIP-1072	Estimate the imaging SE and SP of 123I MIP 1072 compared to 111In capromab pendetide in mPCa
NCT02349022	Phase 2	[89Zr]Df-IAB2M	Compare SE/SP/PPV/NPV/Accuracy of [111In] capromab pendetide SPECT/CT to [89Zr]-Df-IAB2M PET/CT as confirmed by pathology
NCT02615067	Phase 3	99mTc-MIP-1404 SPECT/CT	Safety and Efficacy of 99mTc-MIP-1404 SPECT/CT Imaging to Detect Clinically Significant PCa in Men With Biopsy Proven Low-Grade PCa Candidates for AS (proSPECT-AS)
ACTRN12617000005358	Phase 2	Ga-68 PSMA-PET/CT	Compare the diagnostic accuracy of Ga-68 PSMA-PET/CT to that of conventional imaging for detecting nodal or distant metastatic disease.

Source: https://clinicaltrials.gov.

*PSMA, Prostate specific membrane antigen; RP, radical prostatectomy; MDX1201-A488, Anti-PSMA monoclonal antibody; PCa, prostate cancer; SE, sensitivity; SP, specificity; BCR, biochemical recurrence; CRPC, castration resistant prostate cancer; GS, Gleason score; PPV, positive predictive value; 123-I-MIP-1072, MIP 1404, MIP-1405, small molecule inhibitor of PSMA; PLND, Pelvic Lymph Node Dissection; NPV, negative predictive value; AS, active surveillance*.

As for the PSMA inhibitor 18F-DCFPyL (2-(3-{1-carboxy-5-((6-((18)F)fluoro-pyridine-3-carbonyl)-amino)-pentyl}-ureido)-pentanedioic acid), Chen et al. evaluated its use in immunocompromised mice utilizing isogenic PSMA PC3 PIP and PSMA- PC3 flu xenografts, suggesting that this agent could be viable and effective in this setting (32). A phase I study is now assessing the biodistribution and pharmacokinetic of 18F-DCFPyL in patients with advanced PCa (NCT02151760).

The early distinction between local disease and metastasis is crucial in the management of patients with PCa. 18F-choline can distinguish lesions with moderate to good sensitivity, but its activity is limited to patients with a PSA >1 ng/mL (39). The results obtained by ^68^Ga-labeled PSMA inhibitors showed a high potential in the detection of small recurrent PCa lesions in patients with low levels of serum PSA (40–42). Indeed, ^68^Ga-labeled PSMA inhibitors are characterized by accumulation in small metastatic deposits and a rapid clearance from the tissue in the background (43). Recently, a comparison between PET/CT and PET/MRI hybrid systems using a ^68^Ga-labeled PSMA compound for the detection of recurrent PCa has been performed. The results showed that Ga-PSMA PET/MRI was far more accurate in the detection of PCa and, at the same time, associated with lower radiation exposure (34).

Beyond ^68^Ga-labeled compounds, 99mTc-labeled inhibitors of PSMA have shown great promise in the detection of PCa lesions. Presently, a phase II study is testing 99mTc-MIP-1404 PSMA inhibitor in patients with high-risk PCa scheduled for radical prostatectomy (RP) surgery including extended pelvic lymph node (LN) dissection compared to histopathology (NCT01667536). Results are expected from the completed phase 3 trial proSPECT-AS (NCT02615067). Primary outcome measures of the study are sensitivity and specificity of 99mTc-MIP-1404 SPECT/CT image assessments to correctly detect clinically significant prostate cancer when compared to histopathology following either RP or prostate biopsy in men with newly diagnosed PCa whose biopsy indicates a histopathologic Gleason Score of ≤ 3 + 4.

Furthermore, BAY1075553 [2-PMPA analogs (2*S*, 4*S*)-2-18F-fluoro-4-(phosphonomethyl) pentanedioid acid] has demonstrated high uptake in PSMA^+^ LNCaP tumor xenografts (44). The phase I study showed that BAY1075553 was able to detect primary PCa, lymph node and bone metastases, although its high uptake with degenerative bone lesions may limit its use in assessing bone disease (45).

Worth mentioning is the registrational phase II/III OSPREY study (NCT02981368) that evaluated the diagnostic accuracy of 18F-DCFPyL PET/CT relative to histopathology, for detecting PCa in pelvic lymph nodes in patients with high risk localized prostate cancer who are planned for RP with lymphadenectomy, and in patients with locally recurrent or metastatic disease willing to undergo biopsy.

### Imaging at Diagnosis of PCa

**Figure 2:**
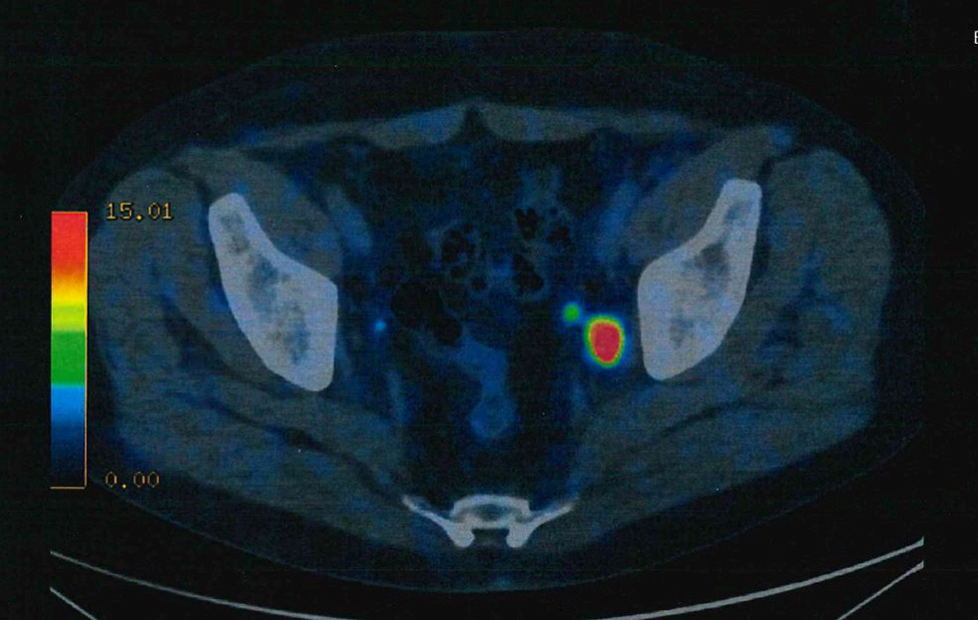
^68^Ga-PSMA ligand PET/CT exhibits solitary left iliac radiotracer-positive lymph node.

A number of recent studies has dealt with the use of PSMA-based imaging for the purpose of diagnosing primary PCa (Figure 2).

Fendler et al. assessed the accuracy of ^68^Ga-PSMA-11 PET/CT in identifying PCa at the initial diagnosis in men with biopsy-proven PCa (46). They found that the optimal SUV_max_ cutoff for distinction of histopathology-positive segments from histopathology-negative segments is of 6.5. With this approach they obtained 67% sensitivity, 92% specificity, 97% positive predictive value, and 72% accuracy.

Woythal et al. (47) evaluated the association of intraprostatic ^68^Ga-PSMA PET/CT features and PSMA immunohistochemical expression in 31 patients who underwent RP and preoperative ^68^Ga-PSMA-11 PET/ CT. ^68^Ga-PSMA-11 PET/CT demonstrated sensitivity and specificity of 87 and 97%, respectively, in the detection of PCa. However, there was no correlation between Gleason Score (GS) and the SUV_max_ of PCa.

On the other hand, Uprimny et al. (48) found that PCa with a GS of 6, 7a (3 + 4) and 7b (4 + 3) showed lower ^68^Ga-PSMA-11 uptake, with SUV_max_ of 5.9, 8.3, and 8.2, respectively, compared to men with a GS greater than 7 (median SUV_max_: 21.2). In addition, men with a PSA of 10.0 ng/mL or above it showed a greater uptake than those patients with PSA levels below 10.0 ng/mL.

The correlation of intraprostatic PSMA uptake with clinical parameter, such as PSA value, GS and d'Amico risk score, was analyzed by Koerber et al. in 104 patients with newly diagnosed PCa (49). Results of this study indicated that men with higher PSA, higher d'Amico risk score and higher GS had greater intensity of PSMA uptake on PET/CT.

The comparison between the multiparametric Magnetic Resonance Imaging (mpMRI) and ^68^Ga-PSMA-11 PET/CT findings showed a concordance in the detection of intraprostatic tumor lesions, with the highest GS of 89.55%. By giving additional molecular imaging information to the mpMRI features, this method can be improved to avoid false-negative results or understaging tumors, in particular the detection of those with the highest GSs. In addition, PSMA PET/ MRI may prove useful in finding lower rates of indolent cancer detection and a great number of intermediate- and high-risk tumors.

### Imaging at Staging of PCa

For pre-operatory staging, current guidelines recommend at least abdomino-pelvic cross-sectional imaging (MRI or CT) and a bone scan, for intermediate- and high-risk PCa (50) only. In a prospective study 30 patients with intermediate- and high-risk PCa underwent preoperative 68Ga-PSMA PET/CT followed by RP and extended pelvic LN dissection. Using pathology as reference, 68Ga-PSMA PET/CT showed a sensitivity of 64% for the evaluation of LN metastasis, with a 95% specificity, 88%, positive predictive value, and 82% negative predictive value (51).

In a prospective, phase II, single center study, Gorin et al. analyzed the diagnostic value of PSMA targeted ^18^F-DCFPyL PET/TC in the preoperative staging of 25 patients considered to be at high risk for having metastatic PCa, despite a negative conventional staging result. With this technique, they obtained a sensitivity and specificity of 71 and 88%, respectively, per patient analysis and 66 and 92% per LN packet analysis (52).

The retrospective study conducted by Maurer et al. (53) involved a 130 men with intermediate and high risk PCa staged with ^68^Ga-PSMA-PET/magnetic resonance tomography or PET/CT. The sensitivity, specificity and accuracy of ^68^Ga-PSMA-PET were 65.9, 98.9, and 88.5%, and those of morphological imaging were 43.9, 85.4, and 72.3%, respectively. Such figures are higher than those for traditional imaging techniques and other alternative PET tracers. Hence, the addition of ^68^Ga-PSMA PET to traditional approaches has the potential to replace current standard imaging, enabling more complete and accurate primary staging.

### Imaging at Biochemical Recurrence of PCa

In men with biochemical recurrence (BCR) after RP or radiotherapy the detection rate of ^68^Ga-PSMA PET/CT increases with higher pre-scan PSA value. In the post-RP patients the rate of ^68^ Ga-PSMA PET/CT was 11.3, 26.6, 53.3, 79.1, and 95.5% for serum PSA levels of 0.01 to <0.2 ng/mL, 0.2 to <0.5 ng/mL, 0.5 to <1 ng/mL, 1 to <2 ng/mL, and ≥ 2 ng/mL, respectively. In the post-radiotherapy patients, the rate was 33.3% for PSA 0.01 to <0.5 ng/mL, 71.4% for PSA 0.5 to <1 ng/mL, 93.3% for PSA 1 to <2 ng/mL, and 100% for PSA ≥ 2 ng/mL (54). Such figures are in agreement with the meta-analysis data by Perera et al. (55). In that study, on per-patient analysis, the sensitivity and specificity of 68Ga-PSMA-11 PET were both 86%. On per-lesion analysis, the sensitivity and specificity were 80 and 97%. ^68^Ga-PSMA PET positivity increased with a shorter PSA doubling time.

Higher rates have been reported by Raucher et al. (56) in a cohort of men with PSA value between 0.2 and 1 ng/ml after RP. The rate of detection was 55% in men with “very low” serum PSA (0.2–0.5 ng/ml) and of 74% in patients with “low” PSA (0.5–1.0 ng <ml). In such investigation the most relevant predictors for 68GaPSMA-ligand PET/CT positivity in multivariable analysis were concurrent androgen deprivation therapy and serum PSA value. Identification of the sites of recurrent disease is of great importance, thus avoiding unnecessary localized treatments in patients of systemic recurrence and avoid the side effects of systemic treatments in men with localized recurrence (57, 58).

Table 1 summarizes the completed trials on PSMA and imaging. For additional trials please visit: https://clinicaltrials.gov.

^18^F-fluciclovine (Axumin^®;^) (18F-FACBC) is an amino-acid targeting radiotracer and not a PSMA based PET/CT agent (59). The sensitivity of 18F-fluciclovine PET for identifying recurrent disease changes with PSA levels, with reported detection rates in the post-prostatectomy biochemical failure setting of 72.0% (for PSA values of less than 1 ng/mL) 83.3% (for PSA 1-2 ng/mL), and 100% for PSA levels of 2 or more ng/mL (60). In patients with pathologically enlarged lymph nodes, presence of true-positive lesions was noted in 29% patients with 18F-fluciclovine vs. 7% patients with CT (61, 62). A prospective study compared overall detection rate of 18F-FACBC and 11C-Choline PET/CT on 28 patients with biochemical relapse after RP. Anti-3-18F-FACBC PET/CT detected 60% additional tumor lesions including 5 (17.8%) additional patients (63).

### PSMA-Targeting Strategies for PCa Therapy

PSMA has been widely utilized as a target antigen due to its constitutive or induced internalization property as well as to its high expression in PCa. Several strategies, including peptides, monoclonal antibodies and aptamers, have been utilized as nanoparticles or prodrugs to improve targeting efficiency in PCa cells. The discovery and development of anticancer aptamers may prove to be relevant contribution to PCa molecular imaging.

Aptamers are short DNA, RNA or peptide oligomers able to assume a specific and stable three-dimensional shape *in vivo* (64). Their high affinity and specificity, similar to antibodies, is achieved by a three-dimensional conformation complementary to the target surface. At this regard, Lupold et al. identified two RNA aptamers (A9 and A10) characterized with high binding affinity to PSMA, leading to the inhibition of its NAALADase/glutamate carboxypeptidase II activity (65). Successively, Xu et al. conjugated A10 aptamer on the surface of micelles, showing high drug uptake in PSMA^+^ cancer cells both *in vitro* and *in vivo* investigations (66).

PSMA can be used as target for delivery of therapeutic agents such as in antibody-drug conjugated (ADC) therapy. PSMA ADC is a fully human anti-PSMA monoclonal antibody conjugated to monomethyl auristatin E through a valine-citrulline linker.

Wang and his group assessed the antitumor activity of PSMA ADC in PCa cell lines *in vitro* and in a novel *in vivo* model of taxane-refractory human PCa. They observed that *in vitro* cytotoxic activity was efficient for PCa cells with increased PSMA expression (>105 molecules/cell; IC50 0.022 nmol/L). In addition, PSMA ADC showed high *in vivo* activity in treating xenograft tumors that have progressed on previous docetaxel therapy (67).

Petrylak et al. (68) reported data from a phase II trial based on PSMA-ADC at 2.5 mg/kg in patients with taxane-refractory metastatic castration-resistant PCa (CRPCa). Thirty-Nine Percent of the patients had been treated with both cabazitaxel and docetaxel, while 58% had received both enzalutamide and abiraterone. Dosing was started at 2.5 mg/kg and adjusted at 2.3 mg/kg for tolerability. The study demonstrated that PSA decline of 30% or more was observed in 36% (2.3 mg/kg) and 16% (2.5 mg/kg). Circulating tumor cell (CTC) decline of ≥50% was seen in 74% patients in both 2.3 and 2.5 mg/kg. Duration of therapy on 2.3 mg/kg was far longer than on 2.5 mg/kg, as well as the rate of serious adverse events (37 vs. 59%). Notably, PSA and CTC decline was associated with higher PSMA expression + CTC level, while PSA responses alone were correlated with lower neuroendocrine (NE) marker expression, thus suggesting that NE differentiation may have a role in this context. On the basis of such results, this study has been further extended (see NCT02020135).

Phage display technology has been used by researchers in the identification of peptide sequences, which can bind to PSMA and, at the same time, inhibit its enzymatic activity. Denmeade et al. conjugated a PSMA-specific peptide to an inhibitor (i.e., Thapsigargin) of the sarcoplasmic/endoplasmic reticulum calcium adenosine triphosphate (SERCA) pump. The type of pump shares the catalytic properties of ion-motive ATPases of the P-type family. It transports calcium ions from the cytoplasm into the sarco-endoplasmic reticulum. Its activity is needed for viability by all types of cells. The conjugate remains inactive until the PSMA-specific peptide is cleaved, thereby starting SERCA inhibition. In xenograft models, thapsigargin induced tumor regression at doses that appeared to be minimally toxic (69). Based on such findings, a phase I study is evaluating the Thapsigargin prodrug G-202 in patients with advanced PCa and other solid tumors (NCT01056029).

PSMA can be used in immunotherapy and radiotherapy approaches. “Adoptive immunotherapy based on infusion of designer T cells engineered with chimeric antigen receptors (CARs)” to potentiate their antitumor activity could serve as a highly specific modality for the treatment of cancer. Thus, PSMA × CD3 diabody is able to retarget human CD4^+^ and CD8^+^ lymphocytes to lyse PSMA-expressing C4-2 PCa cells. Other 1st and 2nd generation anti-PSMA designer T cells have shown their activity in both *in vitro* and *in vivo* studies (70). More recently, mouse-human chimeric IgG1 of 2C9 (KM2777) has been fused with C-terminus interleukin-2 (IL-2). In a xenograft tumor model using PSMA-expressing PCa cells, this fusion, KM2812, showed evident antitumor activity, with complete regression in some cases (71). Bispecific antibodies have been utilized in human clinical trials. A phase I trial has studying the safety of adoptive transfer of autologous T cells targeted to PSMA for the treatment of castrate metastatic PCa patients (NCT01140373). Vaccine is another very important area that utilizes PSMA as a target to increase cellular and humoral immune responses against tumor cells (72).

Concerning the potential role of PSMA targeted antivascular radiotherapy, Bandekar et al. has evaluated liposomes loaded with the α-particle generator 225Ac to kill in a selective manner PSMA positive PCa cells. In such study, anti-PSMA–targeted liposomes have been able to kill PSMA positive cells, including the endothelial cells expressing PSMA, thus suggesting their use for selective antivascular radiotherapy (73).

### Therapy of Metastatic Castration Resistant PCa (CRPCa) With Radiopharmaceuticals

**Figure 3:**
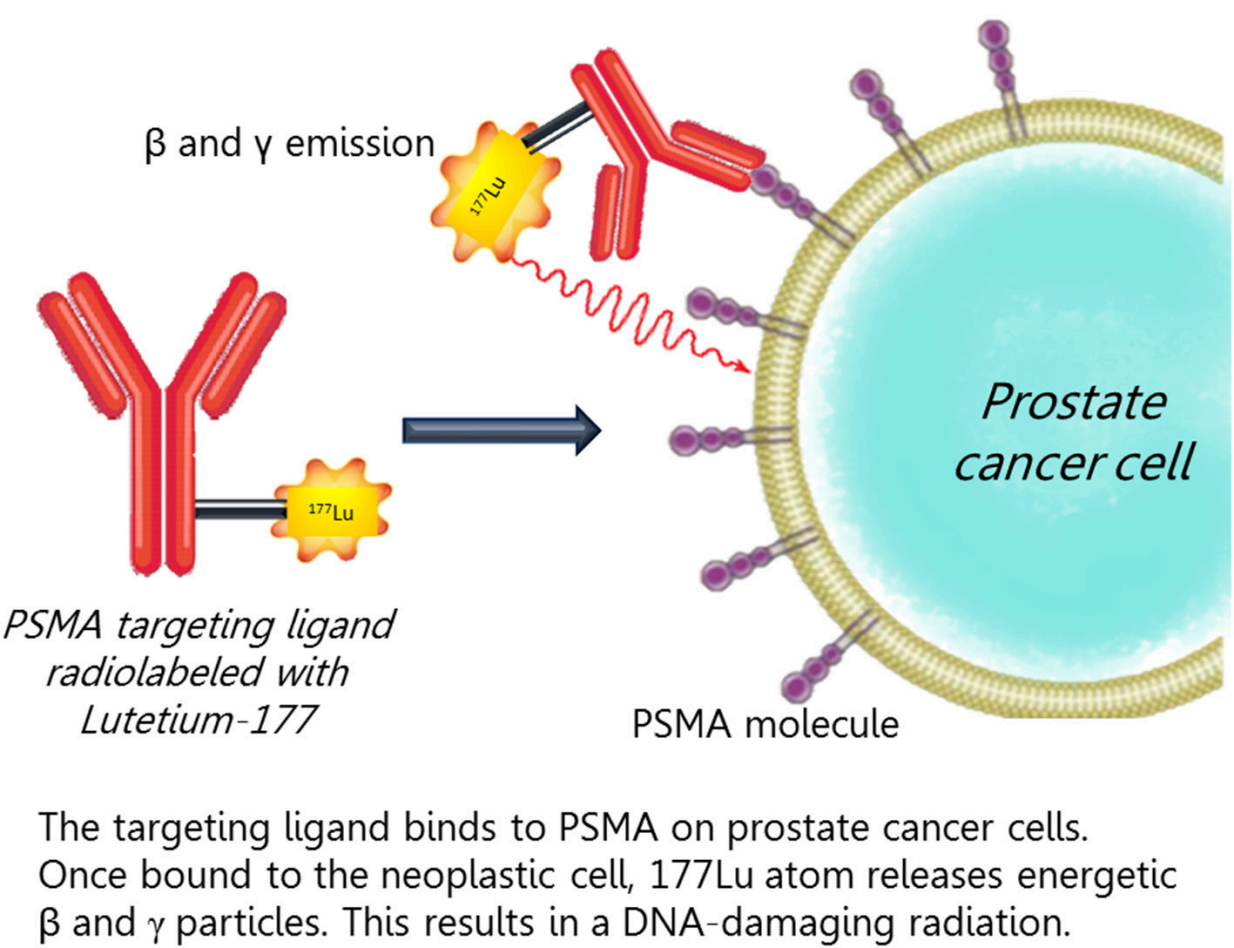
The targeting ligand binds to PSMA on prostate cancer cells. Once bound to the neoplastic cell, 177Lu atom releases an energetic beta and gamma particles that results in a DNA-damaging radiation.

Recently, studies have explored the role of PSMA-based treatments with radiopharmaceuticals of metastatic castration resistant PCa (Figure 3). The first antibody-based radiotherapeutic was Yttrium-90 (90Y) capromab. Phase 1 (74) and Phase 2 (75) studies were unsuccessful for significant toxicity and lack of efficacy.

J591 was the first humanized monoclonal antibody directed against the extracellular domain of PSMA (76). The PSMA antibody–based radiotherapeutic Lutetium-177 J591 (177Lu-J591) showed acceptable toxicity with evident targeting of known metastatic sites in a phase 1 trial. In phase II, almost 60% of men showed a decline in serum PSA levels, 10.6% of them experiencing a >50% decline in PSA (77). Myelosuppression associated with treatment was reversible. “Fractioned dosing allowed for higher cumulative doses with less toxicity” (78). However, there are limitations to the use of PSMA antibody—based radiotherapeutic. Lutetium-177 J591, i.e., slow diffusion of the antibodies into solid tumor lesions and hematotoxicity caused by a long circulation time in the blood (79). Retrospective studies have caveats such as treatments that have been done outside of clinical trials, unrecognized and unmeasured covariates that might influence final results.

A recent systematic review showed that 43% of the men showed a maximum decline of serum PSA of ≥50% following treatment with 177Lu-PSMA radioligand therapy (RLT). In particular, 177Lu-PSMA RLT gave objective remission and decline of PSA of ≥50%, more often than with third-line treatments (enzalutamide and cabazitaxel). Median survival was longer after 177Lu-PSMA RLT than after third-line treatment, the difference being not significant from statistically point of view (80).

A promising available option for patients with mCRPC is the recently investigated compound PSMA–DKFZ-617, a small molecule peptide, rather than an antibody, chemically conjugated with 177Lutetium that binds with high affinity to PSMA. Unlike antibodies such as J591, it shows more rapid plasma clearance, higher affinity binding to PSMA and lower toxicity. Interesting outcomes came up from a single-arm, single-center, phase 2 trial (ACTRN12615000912583) recently published by Hofman et al. (81, 82). Patients with mCRPC and progressive disease after standard treatments underwent screening PSMA and FDG-PET/CT to confirm high PSMA-expression. After four cycles of intravenous [${}^{1^{77}}$Lu]-PSMA-617, 17 (57%) of 30 patients (95% CI 37–75) obtained a PSA decrease of 50% or more, objective response in nodal or visceral disease in 14 (82%) of 17 patients with measurable disease and reported minor toxic effects and improvement in pain severity. Phase 3 trial of 177Lu-PSMA-617 (NCT03511664) is currently recruiting. A multicenter randomized trial comparing LuPSMA with cabazitaxel chemotherapy (NCT03392428) is ongoing.

Table 2 summarizes a selection of active and completed trials on PSMA and therapy. For a full list of trials please visit: https://clinicaltrials.gov.

**Table 2:** Selection of trials of PSMA-based therapy (Selection based on active and completed trials).

**NCT identifier**	**Study phase**	**Drug**	**Study objectives (Number of patients)**	**Study results**
NCT01695044 NCT02020135	Phase 2 (Extension Study)	PSMA ADC	Assess total serum PSA response, CTC response, overall radiologic response in mCRPC pts (119 pts- completed 17 pts) in two groups: (1) CHT-experienced and (2) CHT naïve.	-PSA response: >30% Decrease in PSA: 29% (1); 32% (2). >50% Decrease in PSA: 11% (1); 21% (2). -CTC response >30% Decrease in CTC: 81% (1), 92% (2). >50% Decrease in CTC: 74%(1),85%(2). -Overall radiologic response Stable disease 61% (1),69% (2); Progressive disease: 13% (1), 9% (2); Partial response: 0 (1), 6% (2)
NCT01414283	Phase 1	PSMA ADC	Determine the maximum tolerated dose of PSMA ADC (13 weeks) (52 pts)	No results posted
NCT01414296	Phase 1 Extended 39-Week	PSMA ADC	Safety and tolerability of PSMA ADC as measured by all adverse events in mCRPC patients (10 pts)	No results posted
NCT00705835	Phase 1	Rs-PSMA	Safety, tolerability, and immune response of vaccine therapy with increasing dose levels of rsPSMA protein (14 pts)	No results posted
NCT00015977	Phase 2	PSMA peptide vaccine	Immunization with PSMA peptide vaccine followed by injection of Interleukin-12 in Metastatic PCa patients, determinate disease response (13 pts)	No results posted
NCT01140373	Phase 1	Autologous T cells targeted to PSMA	Safety and tolerability using increasing doses of engineered autologous T cells targeted to PSMA after cyclophosphamide in CMPC patients (13 pts)	No results posted
NCT02202447	Phase 1	EC1169	Safety, pharmacokinetic profile and preliminary efficacy of PSMA Targeting-Tubulysin Conjugate EC1169 in Patients With Recurrent Metastatic CRPC (40 pts)	No results posted
NCT00694551	Not Applicable	Polypeptide vaccines: PSMA27-35-PSMA687-701	Pilot immunotherapy study of combination PSMA and TARP peptide with Poly IC-LC adjuvant in patients with elevated PSA after initial definitive treatment (29 pts)	Adverse events (Grade 3 or higher): 0 pts PSA doubling: 19/29 pts; No PSA doubling: 10/29 pts
NCT00916123	Phase 1	177Lu-J591	Effectiveness of 177Lu-J591 antibody in combination with docetaxel chemotherapy against metastatic CRPC (15 pts)	No results posted

*For a full list of trials please visit: https://clinicaltrials.gov PSMA ADC, Prostate Specific Membrane Antigen Antibody Drug Conjugate; CHT, chemotherapy; PSA, prostatic specific antigen; CTC, Circulating tumor cells; 177Lu- J591, Anti-prostate-specific Membrane Antigen Monoclonal Antibody; CMPC, Castrate Metastatic Prostate Cancer; TARP, T-cell receptor γ alternate reading frame protein; CRPC, castrate-resistant prostate cancer; Rs-PSMA, Recombinant Soluble PSMA*.

## Conclusion

PSMA represents an attractive target for the detection and treatment of patients with PCa. PSMA immunohistochemical evaluation should be further investigated as a predictive marker in men with metastatic PCa, to guide clinicians in the selection of the most appropriate imaging technique and therapy in individual patients. The choice of emerging PSMA-targeted tracers and therapeutic agents requires further investigation in order to identify the most specific compound for the distinct sites and phases of the disease (83–85). As our understanding of the role of PSMA in prostate carcinogenesis advances and molecular techniques become more refined, PSMA-based strategies will have a crucial role in the evolving diagnostic and therapeutic landscape of patients with PCa.

## Author Contributions

RM and MSc conception and design. AC and MSa drafting the manuscript. MC, FM, and AG review of the literature. NB and AL-B critical revision of the manuscript.

### Conflict of Interest Statement

The authors declare that the research was conducted in the absence of any commercial or financial relationships that could be construed as a potential conflict of interest.
